# Do Roux-en-Y gastric bypass patients meet the dietary guidelines?

**DOI:** 10.1186/2049-3258-72-S1-P4

**Published:** 2014-06-06

**Authors:** Ina Gesquiere, Kelly Van Meerbeeck, Veerle Foulon, Patrick Augustijns, Matthias Lannoo, Ann Meulemans, Bart Van der Schueren, Christophe Matthys

**Affiliations:** 1 Department of Pharmaceutical and Pharmacological Sciences, KU Leuven, Leuven, Belgium; 2 Department of Health and Technology, Leuven University College, Leuven, Belgium; 3 Department of Pathology Abdominal Surgery, University Hospitals Leuven, Leuven, Belgium; 4 Clinical and Experimental Endocrinology, KU Leuven/University Hospitals Leuven, Leuven, Belgium

## Background

The prevalence of obesity has increased to epidemic proportions and, as a result, the number of bariatric surgeries has increased worldwide. To date, bariatric surgery remains the sole medical intervention that achieves considerable and sustained weight loss. As both obesity and bariatric surgery are associated with nutritional deficiencies, the aim of this study was to evaluate the dietary intake of macro- and micronutrients in patients before and after Roux-en-Y gastric bypass (RYGB).

## Methods

A prospective observational study was performed at University Hospitals Leuven, Belgium. Patients completed a dietary record of two non-consecutive days before RYGB and 1 and 3 months after RYGB. Intake of macronutrients and micronutrients was calculated for the different time-points. Paired sample t-tests were performed to analyse differences between time-points.

## Results

**Table 1:** Intake of macronutrients at different time-points, shown as mean±SD.

n=22	Intake pre-RYGB	Intake 1 month post-RYGB	Intake 3 months post-RYGB	Significance
**Carbohydrates (g)**	245.2±72.4	81.8±39.1	110.9±51.42	1,2

**Proteins (g)**	87.3±23.8	37.2±16.6	48.0±14.4	1,2,3

**Fat (g)**	92.2±40.4	20.5±12.6	36.3±16.2	1,2,3

**1** p<0.01:pre-op vs post-op 1 month; **2** p<0.01:pre-op vs post-op 3 months; **3** p<0.01:post-op 1 month vs post-op 3 months

**Table 2:** Intake of micronutrients at different time-points, shown as mean±SD.

	Intake pre-RYGB (32 patients)	Intake 1 month post-RYGB (28 patients)	Intake 3 months post-RYGB (26 patients)	Significance
**Ca (mg)**	970.4±519.6	638.4±287.9	695.1±352.3	

**Fe (mg)**	12.6±3.7	5±2.9	6.0±1.8	1,2

**Cu (mg)**	2.1±1.5	1.0±0.9	4.9±18.6	

**Zn (mg)**	46.6±92.1	10.2±21.1	6.6±3.7	

**Vitamin A (µg)**	962.8±405.2	721.5±490.0	787.5±716.6	

**Vitamin B1 (mg)**	1.7±0.7	0.6±0.3	0.8±0.3	1,2

**Vitamin B12 (µg)**	5.4±2.5	2.3±1.5	3.3±1.8	1,2

**Vitamin C (mg)**	138.9±83.8	70.3±56.7	85.1±52.2	1,2

**Vitamin D (µg)**	8.4±5.1	5.2±3.3	4.2±3.2	

**1** p<0.01:pre-op vs post-op 1 month; **2** p<0.01:pre-op vs post-op 3 months; **3** p<0.01:post-op 1 month vs post-op 3 months

## Conclusions

The intake of macro- and micronutrients is markedly decreased one month after RYGB. At three months post-surgery, the intake of macronutrient increases, but the micronutrient intake remains identical at a worryingly low level. Our data clearly suggest that nutritional guidance is essential following bariatric surgery.

## Trial registration

Clinicaltrials.gov #NCT01571180.

